# Do Flower-Rich Windbreak Margins Enhance Predator Communities in Citrus Orchards?

**DOI:** 10.3390/insects17101036

**Published:** 2026-10-09

**Authors:** Romain Exilien, Angela Chuang, Brett Blaauw, Gary Knox, Tracy Liesenfelt, Iris Strzyzewski, Kathi Malfa, Lauren Diepenbrock, Xavier Martini

**Affiliations:** 1 Department of Entomology and Nematology, North Florida Research and Education Center, University of Florida, Quincy, FL 32351, USA; romainexilien@ufl.edu (R.E.);; 2 Department of Environmental Sciences and Studies, Department of Biology, Washington College, Chestertown, MD 21620, USA; achuang2@washcoll.edu; 3 Department of Entomology, University of Georgia, Athens, GA 30602, USA; 4 Department of Environmental Horticulture, North Florida Research and Education Center, University of Florida, Quincy, FL 32351, USA; 5 Department of Entomology and Nematology, University of Florida, Gainesville, FL 32608, USA; 6 Department of Entomology and Nematology, Citrus Research and Education Center, University of Florida, Lake Alfred, FL 33850, USA

**Keywords:** Asian citrus psyllid, conservation biological control, *Diaphorina citri*, floral resource, floral strips, habitat management, natural enemies, windbreaks

## Abstract

Conservation biological control is central to sustainable pest management; however, its effectiveness depends on providing natural enemies with adequate floral resources and habitat structure. In Florida citrus, living windbreaks offer potential for improvement through floral resource provisioning. We evaluated whether flower-enriched windbreaks increase predator abundance and group richness in citrus orchards in north and central Florida over two years and assessed the role of wildflowers in supporting predator communities. In central Florida, treatment groves supported higher predator abundance and group richness across both sampling methods (vacuum and sticky trap) and both study years, particularly spiders, ants, minute pirate bugs, long-legged flies, and wasps. In north Florida, floral plantings did not significantly increase overall predator abundance or group richness, although some treatment effects emerged in the second year, suggesting delayed benefits related to plant establishment. Predator–plant interactions were region-specific: blanket flower attracted the highest proportion of predator visitors in both regions, accounting for 66% of visits by ants in central Florida and 62% of visits by syrphid flies in north Florida. Predator abundance was generally higher near windbreak edges in both regions. In central Florida, wasp abundance was significantly negatively associated with Asian citrus psyllid abundance, with each one-unit increase in wasp density associated with an approximately 2.7% decrease in expected psyllid abundance, whereas spider and ant associations were not statistically significant. Floral enhancements can strengthen predator communities, but outcomes vary across regions and years and depend on regional conditions, plant establishment, predator functional traits, and ecological interactions. Therefore, conservation biological control in citrus requires region-specific plant selection and consideration of ecological context beyond abundance alone.

## Simple Summary

Citrus growers rely on predators to control pests in the orchards. We tested whether flowering plants in citrus windbreaks could enhance predator abundance and group richness across two Florida regions over two years. Flower plantings increased predators, particularly spiders and ants, in central Florida, whereas responses in north Florida were weaker. Blanket flower attracted the highest proportion of visitors in both regions, accounting for 66% of visits by ants in central Florida and 62% of visits by syrphid flies in north Florida. Wasp abundance was negatively associated with Asian citrus psyllid (ACP) abundance; each one-unit increase in wasp density was associated with an approximately 2.7% decrease in ACP abundance. Spider and ant associations were not significant. Flowering windbreaks can support predators, but regional responses mean growers should select plants suited to conditions.

## 1. Introduction

Conservation biological control, which consists of modifying the environment or management practices to promote natural enemies, is central to sustaining pest suppression in agroecosystems [1,2]. Predatory arthropods play important roles in regulating agricultural pests; however, their effectiveness depends on the availability of shelter, prey, and non-prey nutritional subsidies such as nectar, pollen, and honeydew, which can enhance survival, longevity, and foraging efficiency [3,4,5,6,7,8,9]. In simplified, monoculture-dominated landscapes with limited floral diversity under intensive pesticide use, natural enemy populations and biological control services can decline [10,11,12]. Understanding how resource availability and habitat structure shape predator abundance and diversity is therefore critical for developing effective conservation biological control strategies [13].

Landscape complexity shapes natural enemy diversity and abundance [14]. Diversifying non-crop vegetation can strengthen natural enemy communities and support pest regulation, whereas intensive pesticide use and simplified landscapes with large crop patches can reduce natural enemy abundance and biological control potential [1,6,15,16]. These patterns are consistent with the “enemies hypothesis,” which proposes that diversified habitats support greater natural enemy densities by providing nutritional resources and refuges [17]. However, increased vegetational complexity does not always translate into enhanced pest control, as poorly selected non-crop plants may harbor alternative pests or interfere with predator foraging, potentially weakening top-down control [18,19,20]. Flowering plants provide nectar, pollen, and microhabitats that can sustain natural enemies when prey are scarce [1,21,22,23], making floral provisioning a widely used habitat-management approach for conservation biological control. In perennial cropping systems, flower-rich hedgerows and other vegetated margins can also promote movement of beneficial arthropods into crop interiors, potentially increasing their contribution to biological control [24,25,26,27,28,29].

In citrus agroecosystems, biological control has long been a cornerstone of pest management, with roots in classical successes such as the suppression of cottony cushion scale by the vedalia beetle [30]. Modern citrus IPM increasingly emphasizes conservation biological control to support resident natural enemy communities, including lady beetles, lacewings, hoverflies, minute pirate bugs, and predatory mites [5,31,32,33]. These diverse predator guilds may contribute to pest suppression through complementary feeding niches, while floral resources can enhance natural enemy longevity and fecundity, particularly during periods of prey scarcity [8]. However, the presence or abundance of natural enemies does not necessarily translate into effective pest suppression, as their contributions to biological control depend on their functional roles and interactions within the agroecosystem.

Citrus is an economically vital crop in Florida, supporting a multi-billion-dollar agricultural economy [34], and the state’s industry has long served as a model system for biological control, from parasitoid wasps regulating armored scale insects [35] to entomopathogenic fungi naturally suppressing citrus whiteflies [36,37,38]. More recently, the introduction of the Asian citrus psyllid (ACP), *Diaphorina citri* Kuwayama (Hemiptera: Psyllidae), the vector of *Candidatus* Liberibacter asiaticus and the bacterial pathogen associated with Huanglongbing (HLB, or citrus greening disease), has severely affected citrus production since 2005 and reshaped citrus pest management [39]. Generalist predator complexes can reduce ACP populations in commercial groves [40], underscoring the potential importance of conservation biological control in this system. Naturally occurring predatory arthropods therefore represent an important component of ACP management, highlighting the need to understand how habitat and resource availability influence their abundance, diversity, and potential contribution to pest suppression.

In Florida citrus systems, windbreaks, a specific type of hedgerow used to reduce wind damage and limit pathogen and pest spread [41], are typically ecologically simplified, consisting of a single row of fast-growing, often introduced tree species that provide limited resources for natural enemies [42]. In other cropping systems, structurally complex, multi-species windbreaks have been shown to enhance biodiversity and ecosystem services [43], but whether floral resource enrichment of windbreaks can similarly strengthen natural enemy communities in Florida citrus remains largely unexamined. Although conservation plantings that provide nectar resources for ACP natural enemies have been evaluated in commercial groves [44], how predator groups respond to floral enrichment, how particular floral resources are associated with predator groups, and whether predator abundance is correlated with ACP populations remain poorly characterized, particularly across Florida’s climatically distinct citrus-growing regions. We hypothesized that flower-enriched windbreaks would increase predator abundance and group richness as compared to conventional windbreaks, with responses varying regionally according to floral resource availability, plant establishment, and the composition of dominant natural enemy groups in north and central Florida.

This study evaluated whether flower-enriched windbreaks influence predator community abundance and group richness in citrus orchards in north and central Florida, and whether predator abundance is associated with ACP abundance. Specifically, we quantified predator responses to floral resource provisioning across regions, sampling methods, and years; characterized naturally occurring and planted floral resources and their associations with predator groups; identified floral species associated with natural enemy recruitment; and examined relationships between predator groups and ACP abundance. By comparing responses across regions and sampling approaches, we aimed to determine whether floral enrichment consistently enhances predator communities and to identify floral and ecological factors that may influence its potential contribution to conservation biological control.

## 2. Materials and Methods

### 2.1. Field Sites and Experimental Setup

We conducted this field experiment over two years (approximately May 2021–April 2023) at two grower-cooperator citrus groves: one in northern Florida (Monticello, FL, USA) and one in central Florida (Lake Alfred, FL, USA) (see [45] for the detailed site-selection design; Supplementary Table S1 for full site details). Each grove was bordered by a single, pre-existing windbreak treeline. Within each grove, two sides of this windbreak were designated for the experiment: one side was enhanced with flowering plants (treatment side) and the other was left unenhanced (control side), while ensuring that both sides shared the same soil, cultivar, tree age, and grove management, differing only in windbreak floral enhancement.

The northern Florida site, Sweet Valley Citrus (30°37′38.28″ N, 83°49′54.34″ W), a regional brand developed by the Cold Hardy Citrus Association (CHCA) that supports small-scale citrus production in North Florida, was planted with Satsuma mandarin, spaced 1.5 × 4.5 m across 75 rows of 24 trees each; trees were mature (>4 years old). A cypress windbreak (initial height 2–2.5 m) stood approximately 12 m from the grove. The grove was drip-irrigated (low-volume/microjet system) weekly, with foliar and soil nutrition, including slow-release fertilizers, applied monthly, and fungicides/insecticides applied preventively against fungal diseases and citrus leafminer approximately every three weeks. In central Florida, all research was performed at a local grower’s orchard in Lake Alfred (28°06′28.6″ N, 81°41′07.8″ W). Trees were mature (>5 years old) in a managed sweet orange orchard bordered by a single row of mature eucalyptus trees (8–25 m tall), which served as the windbreak. Experimental plots were established adjacent to this windbreak, with each treatment extending across five citrus rows separated by two buffer rows; row middles were spaced 6 m apart, with 3 m between trees within a row. The grove was irrigated via a low-volume/microjet system scheduled according to local weather conditions, with plant nutrition maintained through an integrated program that included slow-release fertilizers. Insecticide applications were made 4–6 times per year, primarily during citrus flushing periods when psyllid activity is highest.

On the treatment side of each grove, we established equal-sized plots (7 × 1.5 m) approximately 2 m from the windbreak treeline on the west-facing side, with each treatment extending across five citrus rows separated by two buffer rows in both North and Central Florida. These plots served as the true experimental unit for the floral-resource treatments, randomly assigned to either four (North Florida) or five (Central Florida) treatment groups, each replicated four times. In North Florida, the four treatments were: (1) flowers (blanket flower and purple coneflower); (2) vine (coral honeysuckle); (3) all (flowers + vine); and (4) an unenhanced control. In Central Florida, a fifth structural layer was added, resulting in five treatments: (1) Flower (blanket flower); (2) Vine (coral honeysuckle); (3) Shrub (buttonbush/American beautyberry); (4) All (flower + vine + shrub); and (5) an unenhanced Control (see Supplementary Table S1 for treatment details per site). The control side of each grove was not subdivided into plot-level treatments; instead, both the treatment and control sides were monitored monthly at rows 1, 3, and 6 (see Section 2.2), providing multiple within-side samples for the side-level comparison. However, because each grove contributed only one treatment side and one control side, this side-level contrast is not replicated at the grove level and is treated as an exploratory, paired comparison distinct from the replicated plot-level analysis.

We began setting up the experiment in October 2020. Prior to planting, Roundup herbicide (active ingredient: glyphosate) was applied once to a 2–3 m-wide strip extending from the windbreak to reduce competition from naturally occurring vegetation. We also applied a thin layer of pine needle mulch around the wildflowers and covered the rest of the plot on the planting date. The wildflower treatments were chosen based on differences in vegetative structure, with a representative herbaceous, vine, and shrub species for each treatment. In addition to nectar accessibility, flowering phenology, and structural arrangement, we prioritized the following characteristics when choosing treatment species: (1) native to Florida to avoid landscape invasion; (2) adapted to the Florida climate and agricultural habitats; and (3) relatively inexpensive and low maintenance to encourage grower adoption.

Plant establishment varied across years and locations due to differential survival and site changes; full details on species, treatment role, planting dates, and densities are provided in Supplementary Tables S2 and S3. We initially planted purple coneflower (*Echinacea purpurea* (L.) as the herbaceous species (12 plants per plot), coral honeysuckle (*Lonicera sempervirens* L.) as the vine species (7 plants per plot), and buttonbush (*Cephalanthus occidentalis* L.) as the shrub species in November 2020 (12 plants per plot). The North Florida sites did not contain a shrub treatment, given the smaller size of the windbreak. Unintended outcomes from the first year required adjustments to field design; buttonbush did not survive the dry spring in 2021, which was not apparent until well into the summer. We kept the dead, woody structures in the plots to determine whether predatory arthropods respond to this additional habitat structure. In January 2022, we introduced American beautyberry (*Callicarpa americana* (L.)) as the new shrub species in the second year (12 plants per plot). Since purple coneflower also did not establish in central Florida following the November 2020 planting, we added blanket flower (*Gaillardia pulchella* Foug.) as an herbaceous wildflower species to both north and central Florida in January 2021 (12 plants per plot). Coral honeysuckle was slow to develop in year 1, so we doubled the density of coral honeysuckle plants for each corresponding treatment to 14 plants per plot in 2021.

Between years 1 and 2, in December 2021, our grower collaborator in central Florida repurposed the grove used as the treatment grove and removed all citrus trees. In year 2, we therefore conducted the experiment in a new grove with similar characteristics and under the same management conditions (Supplementary Table S1), where all floral treatments (American beautyberry, coral honeysuckle, and common blanket flower) were replanted. Therefore, the 2021–2022 observations at this location represent data collected from two comparable but spatially distinct groves rather than a repeated measurement of the same experimental site.

To account for the impact of naturally occurring flowering species on arthropod abundances and richness, we managed the plots only minimally throughout the two-year sampling period. No direct herbicides or insecticides were applied. We hand-weeded to facilitate plantings only when naturally occurring vegetation threatened the establishment of our treatment species, particularly during the initial three months, and occasionally trimmed overgrown grasses near the plantings. Otherwise, naturally occurring species were allowed to grow in the plots to mimic the low-maintenance conditions of the flower strips that citrus growers would adopt. These species were included in subsequent floral assessments (Supplementary Table S2).

### 2.2. Arthropod Sampling

We sampled and surveyed arthropods monthly at both sites from May through December in each of the two study years (2021 and 2022), for a total of 8 sampling months per year (16 sampling months overall), to track trends in abundance and group richness over time as the wildflower plantings established. We used three different methods to sample a variety of arthropod predators: visual count, vacuum sampling, and yellow sticky traps. (1) Each month, we performed a 5-min visual count of all predators within the treatment plots, slowly walking through each plot to record as many individuals as possible. To minimize variability, all visual counts were standardized to morning hours and conducted under normal weather conditions (i.e., surveys were not performed during rain or high wind). (2) Once a month, we randomly sampled 12 trees per grove for 1 min per sample using vacuum sampling, with four trees sampled from each of rows 1, 3, and 6 [45]. For each minute of vacuum sampling, we aspirated from a randomly selected tree and the two adjacent trees, so each sample represents arthropods found in three trees. Vacuum samples were collected in mesh bags and placed in a bucket with ethyl acetate. Samples were frozen within five hours and held until identification. (3) Once a month, we placed 16 yellow sticky traps per grove, with four each at the floral planting plots by the windbreak, as well as rows 1, 3, and 6. The sticky traps were twist-tied to white step-in fence posts 1 m in height and placed either in the middle of floral planting plots or within 0.15 m of each randomly chosen citrus tree. Each trap was placed in a different treatment plot. The sticky traps were generally collected 14 days from deployment; on a few occasions, traps were collected early to accommodate grower management schedules. To account for possible uneven arthropod counts due to this, we divided the arthropod counts by the number of days each trap was deployed to determine the arthropod density per day. The sticky traps were wrapped with plastic wrap in the field and kept in a freezer to maintain specimens until laboratory identification.

Although we identified all arthropods collected through these techniques to the order level, we focused our analyses on specific predator groups of interest (Table 1). These ranged in different taxonomic levels, from order (e.g., Araneae, or spiders), family (e.g., Syrphidae), genus (*Orius* sp.), or species (e.g., *Chrysoperla rufilabris*), depending on what could be reliably identified through the plastic wrapping of the sticky traps. Our predator groups included the following: long-legged flies (Dolichopodidae), hover flies (Syrphidae), lady beetles (Coccinellidae), lacewings (Hemerobiidae, Chysopidae), wasps, ants (Formicidae), spiders (Araneae), minute pirate bugs (*Orius* sp.), and praying mantises (Mantidae). Although we observed mantids in the field, we ultimately did not find any in our samples, so these were omitted from more detailed analyses. We calculated predator group richness within each sample simply by summing the number of each group present (e.g., a sample containing dolichopodids, spiders, and lacewings would have a richness score of 3).

Asian citrus psyllid (ACP) abundance was quantified from the same vacuum and yellow sticky-trap samples described above, rather than using a separate sampling protocol. Because ACP counts were obtained from the identical samples used to quantify predator abundance, collected from the same trees, traps, and plots on the same monthly sampling dates, ACP and predator observations were inherently matched in space and time. No additional spatial or temporal alignment was therefore required. Only adult ACP were included in the analysis because eggs and nymphs are concealed within developing flush and are not reliably captured using vacuum or sticky-trap sampling.

To determine which floral resources were associated with predator group activity, we quantified predator visitation on both the planted floral strips and naturally occurring flowering species within the citrus orchards. Floral visitation was monitored monthly via 5-min standardized visual inspections of each plot, conducted while slowly walking the plot area to maximize arthropod detection. During each observation period, all arthropods actively visiting flowers were recorded and identified to the lowest possible taxonomic level in the field. Observations focused on arthropods engaged in floral foraging behavior, such as nectar or pollen feeding. Both planted species and spontaneous flowering plants occurring within the groves were included to capture the full range of floral resources available to predators. This approach allowed us to identify predator guilds associated with specific flowering plants and to compare the relative visitation of planted versus naturally occurring floral resources within the citrus system.

### 2.3. Statistical Analyses

We conducted all statistical analyses and data visualization in R version 4.2.0 using R Project for Statistical Computing [58]. Arthropod predator abundance and group richness were analyzed using zero-inflated generalized linear mixed models fitted by maximum likelihood with the package *glmmTMB* [59]. Fixed effects included either grove (treatment vs. control) or plot-level treatment (control, shrub, vine, herb, and all plants), as well as row number (rows 1, 3, and 6) as a proxy for distance from the windbreak. Sampling date was included as a random effect. Because the datasets contained many zeros and overdispersed count data, models were fitted using zero-inflated negative binomial or Poisson distributions, as appropriate. Zero inflation was diagnosed by comparing Akaike Information Criterion (AIC) values between zero-inflated and standard (non-zero-inflated) Poisson/negative binomial model fits, with a zero-inflated structure retained when it improved model fit.

Model diagnostics were evaluated using the *DHARMa* package to assess residual distributions, overdispersion, and potential outliers through simulated residual analyses [60]. Model selection was performed using backward stepwise elimination based on Akaike Information Criterion (AIC) scores [61], beginning with full models that included all fixed effects and the grove × row interaction. Backward elimination followed the principle of marginality: interaction terms were tested for removal, and no main effect was removed from the model while an interaction involving that effect remained in the model if significant (*p* < 0.05). The grove × row interaction was therefore evaluated first, and grove and row were retained as main effects for as long as this interaction remained in the model, regardless of their individual significance. Final models were evaluated using Type III Wald χ^2^ tests. When significant fixed effects were detected, pairwise comparisons were conducted using Tukey’s HSD tests and estimated marginal means with the *emmeans* package [62].

The relationship between predator community and Asian citrus psyllid abundance was analyzed using generalized linear mixed models with a negative binomial distribution and log link. The initial full model included major predator groups, including spiders, ants, wasps, long-legged flies, lacewings, and lady beetles. Final model selection based on AIC retained wasp, spider, ant, and lacewing abundance as predictors. Wasp abundance was significantly and negatively associated with ACP abundance, whereas spider, ant, and lacewing abundance were not statistically significant predictors. Effect sizes were expressed as incidence rate ratios (IRR = exp(β)) with 95% confidence intervals. Marginal effects were visualized using predicted response curves generated with the *ggeffects* package [63].

Vacuum sampling and sticky trap data, both collected from the citrus canopy and windbreak rows (only sticky trap), were analyzed together to assess grove- and row-level predator abundance and group richness. Visual count data, collected exclusively within the floral plots, were analyzed separately to characterize predator visitation and plant–predator associations at the floral resource level.

## 3. Results

### 3.1. Summary of Predator and Pest Abundance by Sampling Technique

Over the two-year sampling period, vacuum sampling captured 2757 arthropods in North Florida and 16,877 in Central Florida, while sticky traps captured 16,186 and 67,071 arthropods, respectively. Predators represented 11.2% (*n* = 310; Supplementary Figure S1a) of vacuum samples in North Florida and 14% (*n* = 2375; Supplementary Figure S1b) in Central Florida, whereas they accounted for 30% (*n* = 4802; Supplementary Figure S1c) and 43% (*n* = 28,752; Supplementary Figure S1d) of sticky trap samples in those respective regions. In vacuum samples, pest taxa, dominated by the Asian citrus psyllid (*n* = 5511), comprised 33.3% (*n* = 5626) of total arthropods in Central Florida and 6.4% (*n* = 176) in North Florida. In sticky trap samples, pest taxa represented 7.3% (*n* = 4873; predominantly ACP, *n* = 2164) in Central Florida and 5.4% (*n* = 877) in North Florida. The proportional composition of predator groups across sampling methods and locations is shown in Supplementary Figure S1, and descriptive summary statistics (totals, means, SEs) for predator abundance are provided in Supplementary Table S4.

### 3.2. Which Floral Planting Treatments Promote Higher Abundance in Windbreaks?

**Figure 1:**
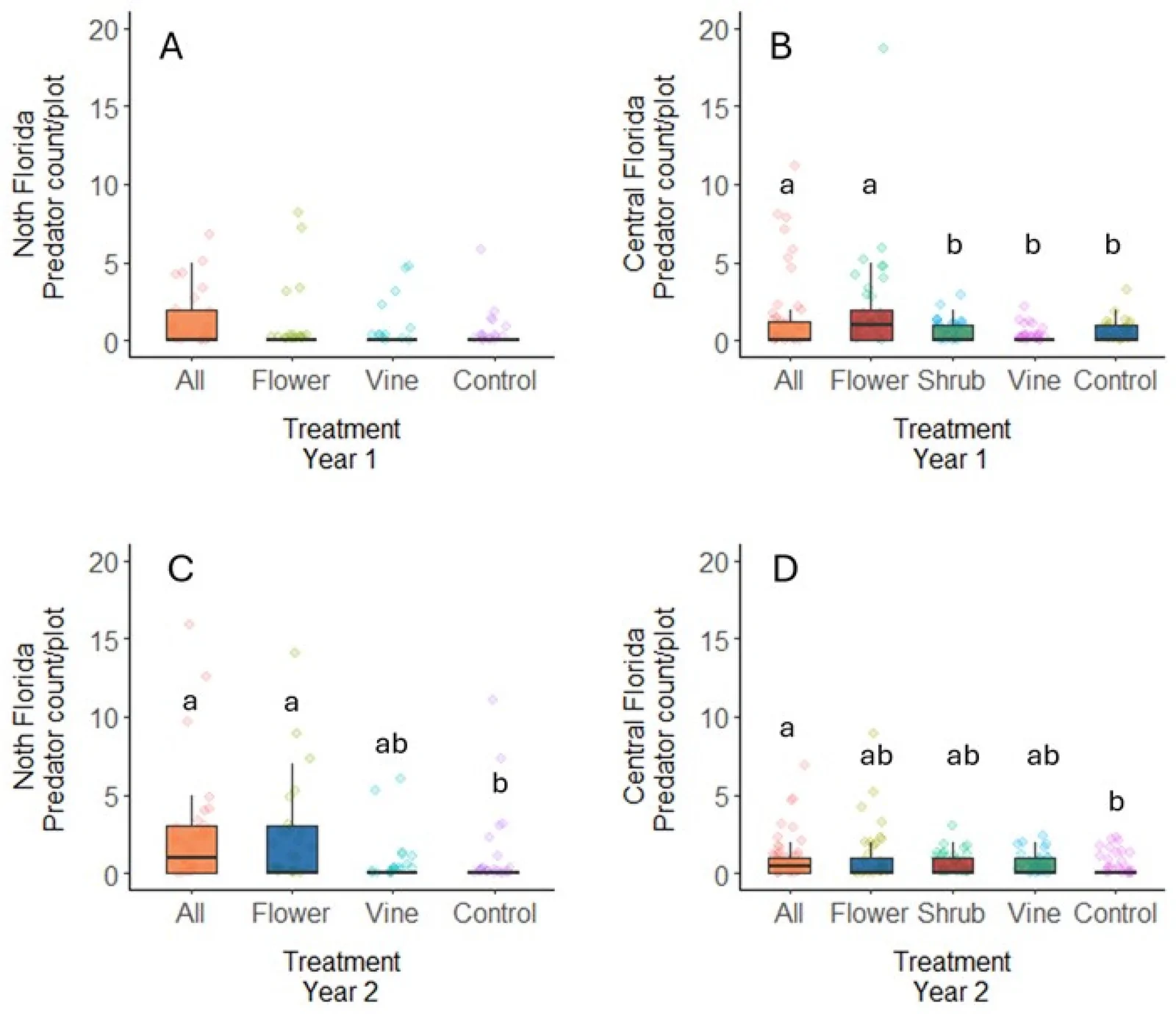
Boxplots with overlaid jittered points showing the total number of predators observed per treatment in Year 1 (2021, (**A**,**B**)) and Year 2 (2022, (**C**,**D**)) in enhanced windbreaks of north (**A**,**C**) (flower: blanket flower and purple coneflower, vine: honeysuckle) and central (**B**,**D**) (flower: blanket flower, shrub: buttonbush/American beautyberry, vine: honeysuckle) Florida. All plants within each plot were sampled (visual count). Boxplots labelled with different letters indicate statistically significant differences.

Predator abundance in this section was assessed using standardized five-minute visual observation counts within each plot. The effectiveness of different windbreak treatments in enhancing predator abundance varied geographically and temporally (Figure 1). In North Florida, during the initial year of species establishment, there were no statistically significant differences in predator abundance among treatment plots (χ^2^ = 3.976, df = 3, *p* = 0.264). By the second year, however, a significant treatment effect emerged (χ^2^ = 14.081, df = 3, *p* = 0.003). Plots with blanket flowers, as well as species mixtures combining blanket flowers, purple coneflower, and honeysuckle, exhibited higher predator abundance as compared to control plots and plots with honeysuckle only (Figure 1A–C). Central Florida, however, demonstrated a more immediate impact, with significant treatment differences observed in both the first (χ^2^ = 43.571, df = 4, *p* < 0.001) and second year (χ^2^ = 13.103, df = 4, *p* = 0.011). In this region, mixed plantings of blanket flowers, American beautyberry, and honeysuckle consistently supported greater predator populations as compared to control and other enhancement treatments (Figure 1B–D).

### 3.3. Do Floral Plantings Increase Total Predator Abundance and Group Richness in Grove-Level Treatments?

Based on our vacuum sampling, we found mixed results on predator abundance and group richness in our treatment vs. control groves across locations (Supplementary Figure S2). In north Florida, predator abundance did not differ between treatment and control groves (χ^2^ = 1.936, df = 1, *p* = 0.164). However, sampling rows had a significant effect. The sixth row had fewer predators than the first and third rows (χ^2^ = 8.975, df = 2, *p* = 0.011; Supplementary Table S3), consistent with a potential influence of windbreak habitats on predator abundance in rows closest to the windbreak, irrespective of the floral treatment. Predator group richness was not affected by grove treatment (χ^2^ = 0.726, df = 1, *p* = 0.394) or row distance (χ^2^ = 3.817, df = 2, *p* = 0.148; Supplementary Figure S2a,b; Supplementary Table S4). In central Florida, predator abundance was higher in treatment groves than in control groves (χ^2^ = 68.719, df = 1, *p* = 0.001), with no effect of row distance (χ^2^ = 0.109, df = 2, *p* = 0.947; Supplementary Figure S2c; Supplementary Table S4). Predator group richness was also greater in treatment groves (χ^2^ = 44.687, df = 1, *p* < 0.001), and row distance had no effect (χ^2^ = 2.137, df = 2, *p* = 0.343; Supplementary Figure S2d; Supplementary Table S4).

**Figure 2:**
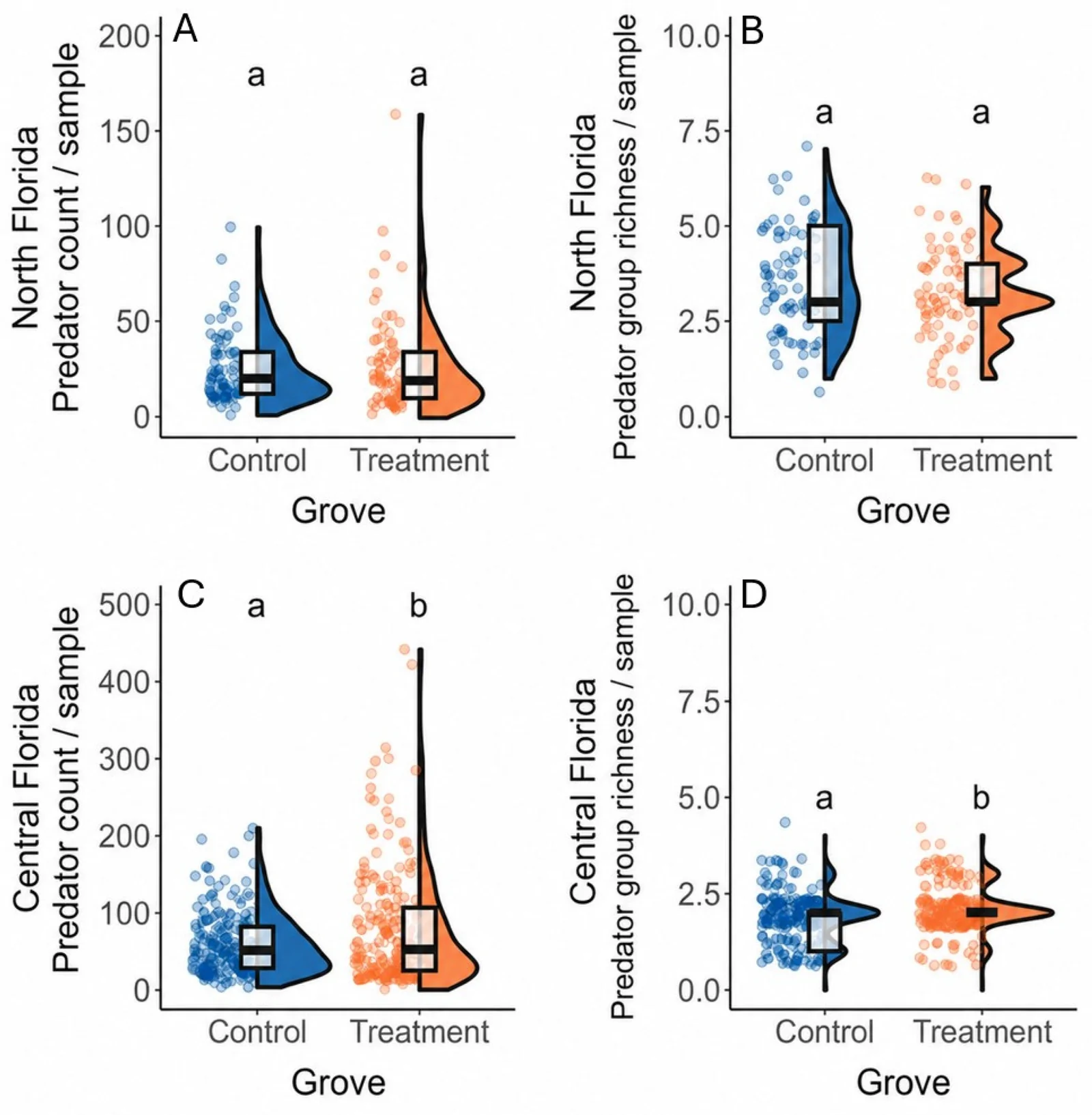
Data points, density kernel plots, and boxplots showing the distribution of (**A**) predator abundance and (**B**) group richness in north Florida, as well as (**C**) predator abundance and (**D**) group richness in central Florida from yellow sticky trap samples. Data points have been jittered to aid in visualization since many values are identical. Different letters indicate statistically significant differences.

Sticky trap data showed similar trends (Figure 2). In north Florida, predator abundance and group richness did not differ between treatment and control groves (abundance: χ^2^ = 1.422, df = 1, *p* = 0.233, and group richness: χ^2^ = 2.435, df = 1, *p* = 0.119; Supplementary Table S4). Traps located in the windbreak captured significantly more individuals than traps placed within the grove rows (χ^2^ = 42.854, df = 3, *p* < 0.001). However, predator group richness was higher at the windbreak and row 1 compared to rows 3 and 6 (χ^2^ = 32.266, df = 3, *p* < 0.001; Supplementary Table S4). In central Florida, grove treatment significantly affected predator abundance (χ^2^ = 15.782, df = 1, *p* = 0.001). Predator abundance also differed by distance from the windbreak, with the highest numbers recorded in row 1, followed by the windbreak plantings, row 3, and row 6 (χ^2^ = 31.720, df = 3, *p* < 0.001; Supplementary Table S4). Predator abundance varied with both grove and distance from the windbreak, as indicated by a significant grove × row interaction (χ^2^ = 13.212, df = 3, *p* = 0.004; Supplementary Figure S3). The treatment grove had higher predator numbers near the windbreak, but this difference levelled off by rows 3 and 6. Similarly, predator group richness was higher in treatment groves (χ^2^ = 20.102, df = 1, *p* < 0.001), but did not vary with distance from the windbreak (χ^2^ = 6.236, df = 3, *p* = 0.101; Supplementary Table S4).

### 3.4. How Do Floral Plantings Influence Individual Predator Groups?

**Figure 3:**
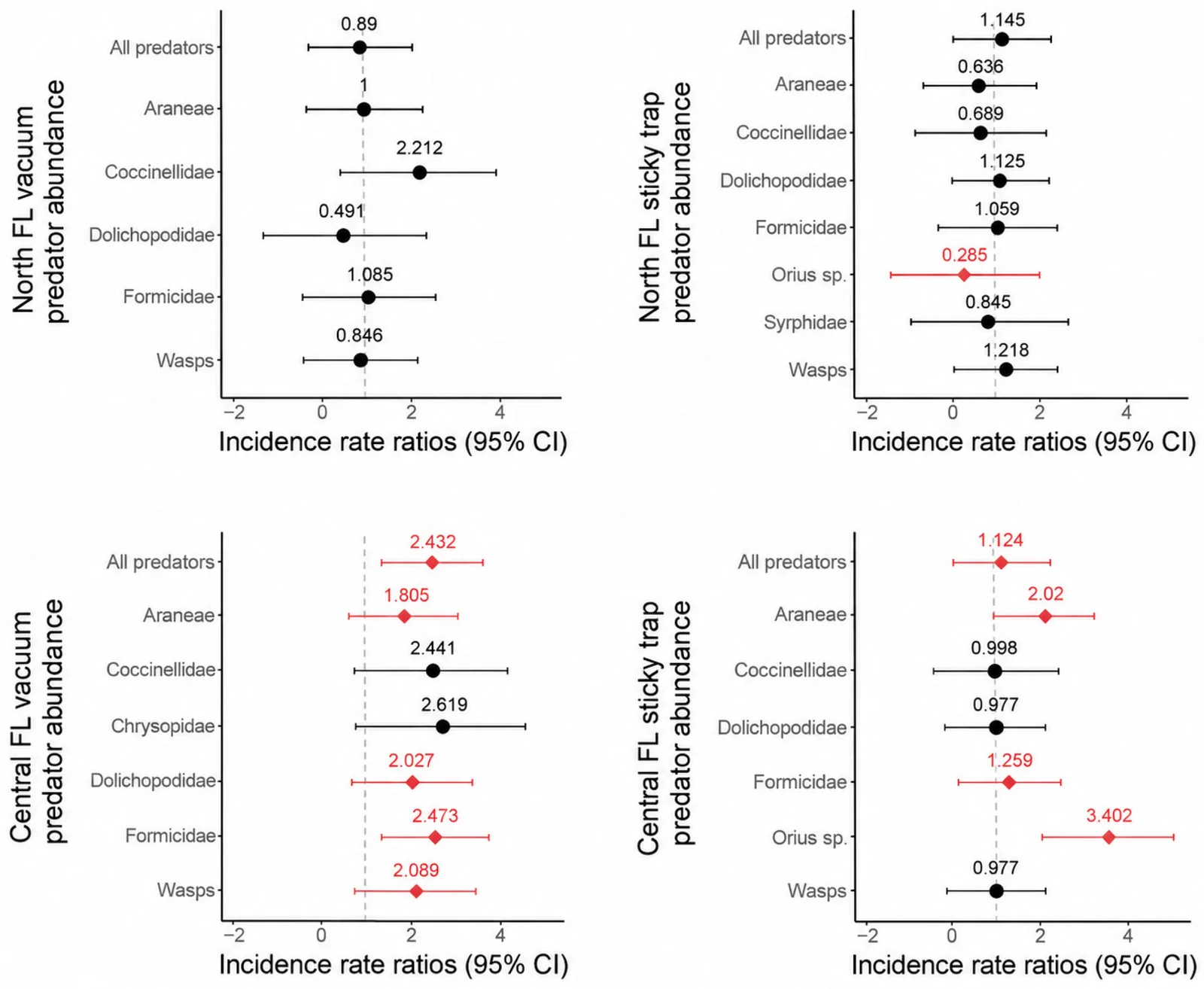
Incidence rate ratios (IRRs) with 95% confidence intervals (error bars) comparing predator group abundance in treatment groves versus control groves, shown separately for each sampling method (vacuum and sticky trap) and location (north and central Florida). IRRs were estimated from generalized linear mixed models (GLMMs). An IRR > 1 indicates higher abundance in treatment groves; an IRR < 1 indicates higher abundance in control groves. Red diamonds denote predator groups for which the treatment effect was statistically significant at α = 0.05. Predator groups with fewer than 10 total individuals across all samples (within a given sampling method and location) were excluded from analysis and are not shown: for vacuum sampling, this applied to Syrphidae (north Florida, *n* = 2; central Florida, *n* = 1), Chrysopidae (north Florida, *n* = 9), and *Orius* sp. (north Florida, *n* = 2); for sticky trap sampling, this applied to Chrysopidae (north Florida, *n* = 2; central Florida, *n* = 3) and Syrphidae (central Florida, *n* = 1).

Predator groups from varying arthropod orders may respond differently to floral treatments; therefore, we further modelled how each individual group responded to grove-level treatment for both vacuum and sticky trap sampling. In north Florida, there was no evidence that any of the groups differed between the treatment and control groves except for *Orius* bugs, which were less common in the treatment grove (Figure 3, Supplementary Tables S5 and S6).

In contrast, in central Florida, we found an overall effect of predators being more abundant in the treatment grove. Spiders, ants, and *Orius* bugs were consistently more abundant in both sampling techniques. Vacuum sampling further suggested that dolichopodids and wasps were more abundant in the treatment grove, although we did not find evidence for this via sticky trap counts (Figure 3, Supplementary Tables S5 and S6).

### 3.5. Which Flowering Plants Are Most Frequently Visited by Predators?

**Figure 4:**
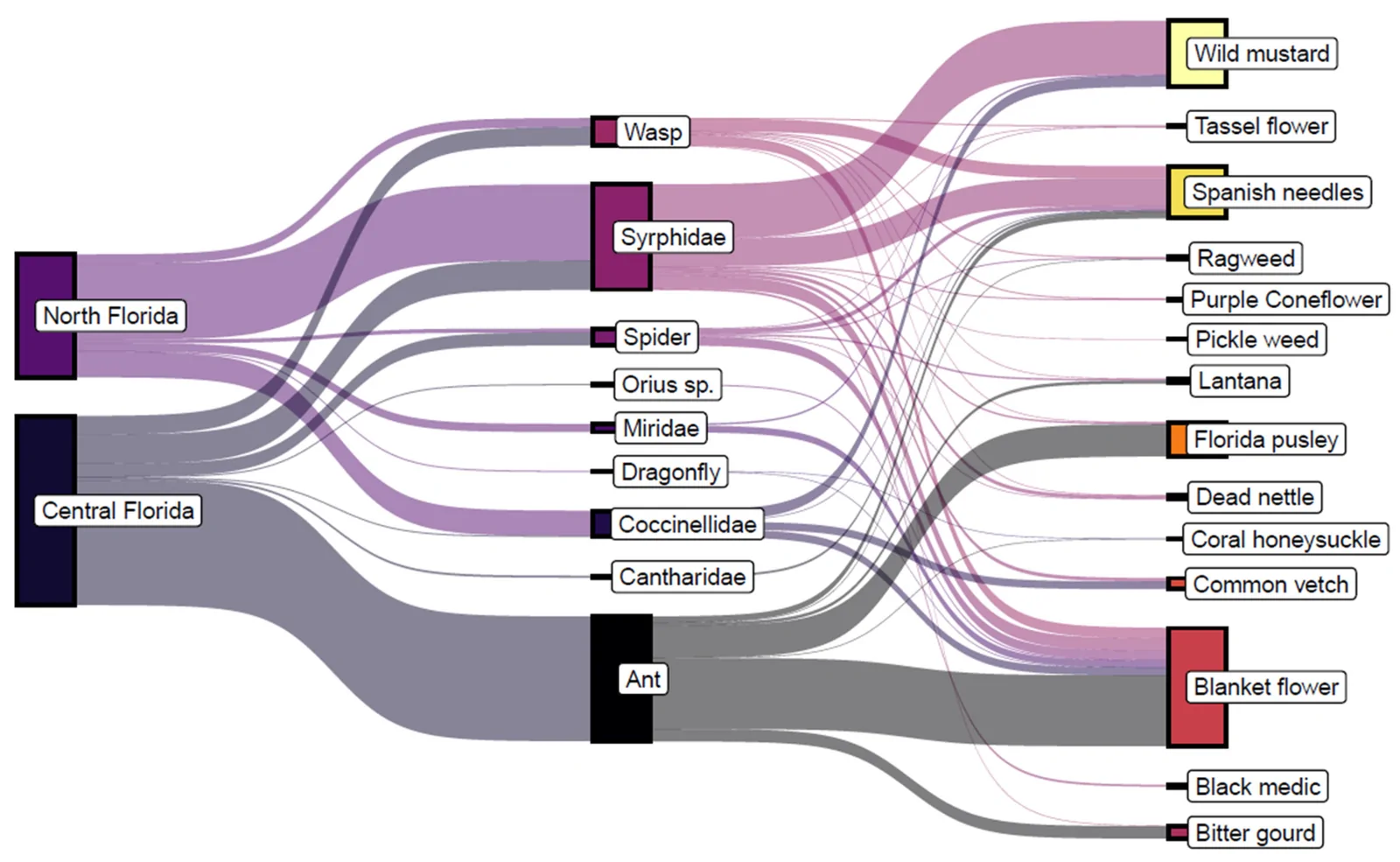
Sankey diagram illustrating associations between flowering plant species and predatory arthropod taxa observed during floral visitation surveys in central and north Florida (2021–2022). Predator taxa are organized by order, with further resolution to family or common name where identifiable.

A total of 660 predator visitation events were recorded across both study sites, with 260 in north Florida and 400 in central Florida (Supplementary Table S7). In north Florida, plant species significantly influenced predator visitation (χ^2^ = 93.635, df = 8, *p* < 0.001). Blanket flower and wild mustard (*Sinapis arvensis* L.), a naturalized common forb, supported the highest levels of predator activity. In this location, the predator community was dominated by flying insects. Syrphidae were the most frequent visitors (62% of observations), followed by Coccinellidae (20%) (Figure 4). The distribution of predator groups differed significantly among taxa (χ^2^ = 48.572, df = 5, *p* < 0.001).

In central Florida, predator visitation differed significantly among plant species (χ^2^ = 70.850, df = 7, *p* < 0.001). Spanish needle (*Bidens alba* L.), a locally common forb, and blanket flower received the highest number of visits and accounted for most predator activity. In contrast to north Florida, community composition in central Florida showed that ants were the dominant visitors, representing 66% of all observations (Figure 4). Their frequency was significantly higher than that of other predator groups (χ^2^ = 85.737, df = 6, *p* < 0.001).

### 3.6. How Is ACP Abundance Associated with Predator Communities?

Assessment of grove-level ACP counts in Central Florida, using both vacuum and sticky trap sampling, revealed significantly higher psyllid counts in treatment grove relative to control grove (χ^2^ = 32.08, df = 1, *p* < 0.0001). Within treatment grove, ACP abundance differed significantly among sampling rows (χ^2^ = 55.162, df = 3, *p* < 0.0001), with lower psyllid counts recorded in windbreak enhancement plots compared to rows 1–6.

**Figure 5:**
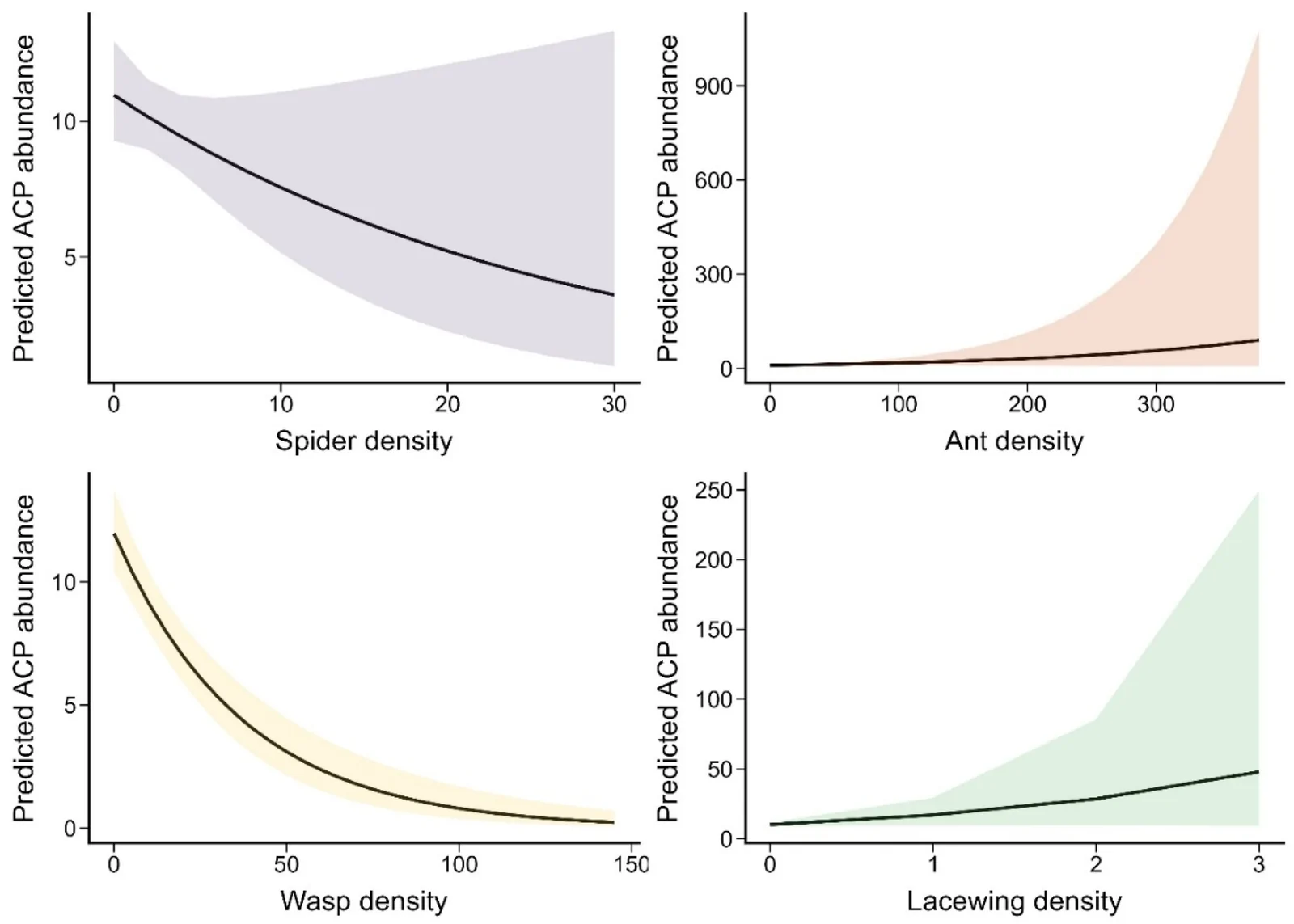
Model-predicted effects of spider, ant, wasp, and lacewing densities on Asian citrus psyllid (ACP) abundance (vacuum and sticky trap sampling).

The relationship between predator community composition and ACP abundance was evaluated using a total of 727 observations. Among the retained predictors, wasp abundance was significantly associated with ACP abundance (χ^2^ = 45.876, df = 1, *p* < 0.001), whereas spider abundance was not (χ^2^ = 2.385, df = 1, *p* = 0.123). The effect size, expressed as incidence rate ratios (IRR), indicated a significant negative association between wasp abundance and ACP abundance (IRR = 0.973, 95% CI: 0.966–0.981). In practical terms, each one-unit increase in wasp density was associated with an approximately 2.7% decrease in expected ACP abundance. Spider abundance also showed a negative but non-significant association with ACP abundance (IRR = 0.964, 95% CI: 0.919–1.010). Ant abundance showed a weak positive but non-significant association with ACP abundance (χ^2^ = 2.875, df = 1, *p* = 0.090; IRR = 1.006, 95% CI: 0.999–1.013), whereas lacewing abundance showed a positive but non-significant association (χ^2^ = 3.330, df = 1, *p* = 0.068; IRR = 1.677, 95% CI: 0.962–2.920). Model predictions showed a negative relationship between wasp density and predicted ACP abundance, with predicted ACP abundance declining as wasp density increased (Figure 5 and Figure S4).

## 4. Discussion

Our study provides partial support for the hypothesis that floral enhancements strengthen predator communities, with strong positive effects in central Florida and weaker responses in north Florida. This reflects clear regional and temporal variation in how floral plantings influence predator abundance. In central Florida, differences among floral treatments were consistent across both years within windbreak plantings. Mixed-species plantings of blanket flower, buttonbush, and honeysuckle consistently supported higher predator abundance, suggesting that diversified floral resources enhance resource continuity and habitat complexity for natural enemies. Overall, predator abundance and group richness were higher in grove treatments across sampling methods, supporting the established role of floral resources in sustaining natural enemies [64,65]. The broad taxonomic range of predatory groups responding positively in central Florida, including spiders, *Orius* sp., dolichopodids, and wasps, further underscores that diverse floral communities can support multiple functional guilds. However, because the Central Florida experiment was relocated and replanted between years, year, site identity, and vegetation establishment were partially confounded. Consequently, the treatment differences observed across both years at this site should not be attributed solely to a first- versus second-year establishment response.

In contrast, higher predator abundance in North Florida was observed only in the second year, consistent with a possible delayed response associated with plant establishment. Blanket flower and mixed plantings, including blanket flower, purple coneflower, and honeysuckle, coincided with predator abundance once vegetation was established. However, during the initial two years, floral plantings did not consistently yield higher predator abundance or group richness relative to control groves. This pattern may reflect the poor establishment of key species, particularly honeysuckle and purple coneflower, which may have resulted in low floral density and inconsistent bloom availability. Additionally, abundant naturally occurring vegetation within North Florida windbreaks may have provided alternative resources, potentially reducing contrasts between treatment and control groves. Because floral density and background vegetation complexity were not directly quantified in this study, these explanations should be considered plausible hypotheses rather than confirmed mechanisms. Similar patterns have been reported where high background resource availability limits detectable responses to habitat manipulations [6]. Habitat enhancements are generally most effective when baseline floral resources are limiting [7,13,66]. A longer establishment period may therefore have produced stronger responses in northern Florida.

Predator abundance increased near windbreaks, a spatial gradient consistent with an edge-mediated effect. This pattern suggests that, even where floral establishment was limited, windbreak vegetation and adjacent plant communities may have functioned as localized refuges or resource areas for predators. Edge-mediated effects on natural enemy distribution are well documented in agricultural landscapes [67,68,69,70], and our results are consistent with these patterns, with grove margins exhibiting greater predator activity than interior rows, although the magnitude of this effect varied by region.

Several ecological factors could contribute to the regional differences observed in predator responses. However, because these variables were not incorporated into our statistical models, their potential influence on the observed trends cannot be confirmed from the present data. For instance, in central Florida, vigorous growth and sustained flowering of *G. pulchella* appeared to provide continuous nectar and pollen resources, whereas colder winter and early spring temperatures in northern Florida may have delayed plant development and flowering, potentially reducing early-season resource availability [45]. Temperature-driven constraints on phenology and floral rewards are well documented in other systems [71,72,73,74,75] and therefore provide a plausible hypothesis for the lower early-season predator abundance observed in North Florida. Differences in background vegetation may also have contributed to these regional contrasts. Northern Florida groves supported abundant resident flora that could have provided alternative nectar, pollen, and habitat structure independently of our treatments. Under such conditions, additional floral strips may provide limited incremental benefits when baseline floral resources are already abundant [6,13]. Regional climatic differences may have also influenced arthropod activity more broadly. Warmer conditions in central Florida are generally associated with longer flowering windows and greater pest and predator activity, whereas cooler conditions in northern Florida may constrain both plant phenology and arthropod activity.

Predator taxa responded differently to floral plantings, reflecting variation in foraging behavior and resource use. In central Florida, spiders, ants, *Orius* sp., dolichopodids, and wasps were consistently more abundant in treated groves. Spiders possibly benefited from increased structural complexity and prey availability near flowering plants [76]. Ants increased in association with floral resources and honeydew-producing insects, consistent with their opportunistic feeding behavior [77,78,79]. *Orius* sp. responses align with known benefits of pollen provisioning for facultative predators [52,80]. Dolichopodids and wasps similarly reflect the importance of nectar resources for supporting predatory Diptera and Hymenoptera.

In North Florida, most predator guilds showed weak or no response to floral enhancement treatments the first year of the experiment, consistent with the combined effects of poor plant establishment and high background vegetation complexity. Predator–plant associations also varied regionally. In central Florida, Spanish needles and blanket flower were the species most frequently visited by ants, whereas in North Florida, wild mustard and blanket flower received the highest visitation by flying predators, particularly syrphids. These regional differences are important because floral enhancement in North Florida was primarily associated with direct foraging by flying predators, whereas in central Florida it was associated with increased visitation by ants, whose mutualistic relationships with honeydew-producing hemipterans may reduce rather than enhance biological control. The identity of dominant floral visitors, therefore, influences whether floral enhancement translates into effective biological control. Syrphids were strongly associated with yellow, pollen-rich flowers, consistent with their nutritional requirements for adult reproduction and longevity [81,82]. Across both regions, blanket flower received the most consistent predator visitation among the species tested, indicating broad attractiveness across taxa. However, visitation frequency alone does not demonstrate that blanket flower provides higher-quality resources or enhances predator fitness, persistence, or pest suppression, as attraction can differ from the actual reward provided by the plant. This attractiveness may also increase unwanted visitors. In central Florida, plots with greater blanket flower abundance had higher ant visitation and were associated with higher ACP densities in treatment groves than in controls.

Contrary to our expectations, predator–ACP associations varied among taxonomic groups in central Florida. Wasp abundance was significantly negatively associated with ACP abundance, with each additional wasp associated with an approximately 2.7% decrease in the expected ACP incidence rate. This pattern is consistent with a potential role of wasps in ACP suppression, although the observational nature of this analysis does not establish a causal relationship or confirm predation. Wasps are recognized predators of ACP and other hemipteran pests [83], and their association with lower ACP abundance warrants further investigation using direct measures of foraging and prey consumption. In contrast, spider abundance showed a weak negative but non-significant association with ACP abundance. Thus, although spider abundance tended to increase as ACP abundance decreased, the evidence was insufficient to conclude that spiders were associated with lower ACP abundance in this system. The observed pattern is nevertheless compatible with previous reports of limited or variable pest-regulatory effects of spiders on hemipteran pests in citrus systems [84]. The positive association observed for ants was also weak and non-significant. Although ants may occasionally prey on ACP eggs or nymphs, they are strongly attracted to honeydew-producing Hemiptera and may protect them from natural enemies in exchange for carbohydrate rewards [85,86,87]. Elevated ant activity has also been associated with reduced predation or parasitism by other natural enemies, including spiders, coccinellids, lacewings, *Orius* spp., and parasitoids [88,89,90]. Thus, even if ant–ACP association could result in the disruption of biological control, the fact that this relationship was not statistically significant suggests that the benefits of planting companion plants are not offset by ant attraction. Lacewing abundance showed a relatively large positive estimated association with ACP abundance with the confidence interval (Supplementary Figure S4). This pattern should therefore be interpreted cautiously and not as evidence that lacewings increased ACP abundance. Rather, the positive direction may reflect a numerical response to greater prey availability or temporal variation in predator and prey populations. Other predators, including lady beetles and syrphids, were not significantly associated with ACP abundance, possibly because of low abundance, temporal variability, or differences in their functional responses to ACP. These taxa nevertheless remain recognized components of the ACP natural-enemy complex with potential contributions to biological control [83].

More broadly, these findings suggest that floral enhancement may increase predator activity without necessarily producing proportional reductions in pest abundance. In central Florida, the greater abundance of blanket flower and associated floral resources coincided with increased arthropod activity, particularly ants, emphasizing the importance of considering the identity and ecological interactions of dominant floral visitors when selecting plants for conservation biological control. Future studies should directly quantify predator foraging, prey consumption, and ACP suppression, particularly for wasps, whose significant negative association with ACP abundance provides a promising hypothesis for further experimental testing, and under conditions where ant activity is experimentally manipulated.

## 5. Conclusions

This study shows that floral enrichment can increase the abundance and taxonomic-group richness of natural enemies in citrus windbreaks, although these effects were context-dependent and were most evident where baseline floral resources appeared to be limited, as in central Florida. Importantly, increases in natural-enemy abundance and richness did not necessarily translate into stronger ACP biological control. Predator–ACP associations varied among taxonomic groups, with wasp abundance showing a significant negative association with ACP abundance, whereas associations involving spiders, ants, and lacewings were not statistically significant. The negative association between wasps and ACP is consistent with a potential contribution to pest suppression; however, direct measures of predation are needed to establish causality. Overall, our findings indicate that floral enrichment should be viewed primarily as a habitat-management strategy for supporting natural-enemy communities rather than as a universally effective approach for conservation biological control. Its effectiveness depends on the identity and functional roles of the taxa recruited, as well as their interactions within the agroecosystem. These findings emphasize that natural-enemy abundance and taxonomic richness should not be assumed to be proxies for biological control. Future research should therefore focus on linking floral-resource enhancement to direct measures of predation, parasitism, and ACP suppression to determine when and under what ecological conditions floral enrichment can provide effective biological control.

## Figures and Tables

**Table 1:** A list of predator groups considered in this study and a non-exhaustive list of citrus pests they are known to be natural enemies of, especially in Florida, with a focus on Asian citrus psyllid.

Predator Group	Scientific Name	Citrus Pests Consumed	References
Hover flies	Diptera: Syrphidae	Asian citrus psyllid adults	[46,47,48]
		Brown citrus aphid	[49]
Long-legged flies	Diptera: Dolichopodidae	Asian citrus psyllid adults	[48]
Minute pirate bugs	Hemiptera: Anthocoridae: *Orius*	Asian citrus psyllid nymphs	[48]
Lady beetles	Coleoptera: Coccinellidae	Asian citrus psyllid nymphs	[46,47,50]
		Brown citrus aphid	[49]
		Hibiscus mealybug	[51]
Lacewings	Neuroptera: Chrysopidae	Asian citrus psyllid eggs and nymphs	
	*Chrysoperla rufilabris*	Asian citrus psyllid eggs and nymphs	[48,52]
		Citrus leafminer	[53]
		Hibiscus mealybug	[51]
	*Ceraeochrysa* spp.	Asian citrus psyllid eggs and nymphs	[48,52]
		Brown citrus aphid	[49]
Spiders	Araneae	Asian citrus psyllid adults and nymphs	[47,48,52]
		Citrus leafminer	[53]
		Hibiscus mealybug	[51]
Wasps	Hymenoptera: Vespidae	Asian citrus psyllid nymphs and adults	[54,55]
Ants	Hymenoptera: Formicidae	Asian citrus psyllid adults	[47,48]
		Asian citrus psyllid nymphs (possibly)	[47]
		Citrus leafminer	[51,56]
Mantids	Mantodea: Mantidae	Asian citrus psyllid adults	[57]

## Data Availability

Data from this study are available upon request to the corresponding author.

## Supplementary Materials

The following supporting information can be downloaded at: https://www.mdpi.com/article/10.3390/insects17101036/s1, Table S1: Details on the sites used, including treatments for each citrus grove; Table S2: Details on the plant species cultivated in this study along with naturally occurring species; Table S3: Plant establishment by species, treatment role, location, year, and planting density; Table S4: A summary of the sums, means, and SEs of arthropod predators found in each location, grove type, and row for vacuum sampling. Note that the overall sums are not comparable between locations due to differences in sampling effort; Table S5: A table of the sums of each group of arthropod predators found in each location, grove type, and row for vacuum sampling. Despite including mantids due to their presence in fields, none were collected via vacuum sampling or sticky traps; Table S6: Estimated parameters, standard errors, z-statistics, and *p*-values for the abundance of each predator group in treatment groves compared with control groves, presented by sampling method and location; Table S7: Floral visitation frequency of predatory/natural enemy arthropod taxa on flowering plant species at Central Florida (CREC) and North Florida (NFREC) field sites; Figure S1: Proportional composition of predatory arthropod groups collected via vacuum (a, c) and sticky trap (b, d) sampling in North Florida (a, b) and Central Florida (c, d) from 2021 to 2023; Figure S2: Data points, density kernel plots, and boxplots showing the distribution of (a) predator abundance and, (b) group diversity in north Florida, as well as (c) predatory abundance and (d) group diversity in central Florida from vacuum samples. Data points have been jittered to aid in visualization since many values are identical; Figure S3: Interaction between grove treatment and distance from windbreak on predicted predator abundance; Figure S4: Incidence rate ratios (IRRs) for the association between natural enemy abundances (wasps, spiders, ants, and lacewings) and Asian citrus psyllid (*Diaphorina citri*) abundance in Central Florida citrus groves. Error bars represent 95% confidence intervals. An IRR > 1 indicates a positive co-occurrence (higher natural enemy counts corresponding to higher ACP counts), whereas an IRR < 1 indicates an inverse association. Among the evaluated groups, wasps showed a statistically significant inverse association with ACP abundance (IRR = 0.973, 95% CI: 0.966–0.981, *p* < 0.001). Spiders (IRR = 0.964, 95% CI: 0.919–1.010, *p* = 0.123), ants (IRR = 1.006, 95% CI: 0.999–1.013, *p* = 0.090), and lacewings (IRR = 1.677, 95% CI: 0.962–2.920, *p* = 0.068) showed no statistically significant associations with ACP counts at the α = 0.05 level.
