# Supplementary material for: Do Flower-Rich Windbreak Margins Enhance Predator Communities in Citrus Orchards?
Source: Insects. 2026 Oct 9;17(10):1036. doi: 10.3390/insects17101036 (PMC13705309; doi:10.3390/insects17101036)
Supplement: Supplementary file 1 [file insects-17-01036-s001.zip › insects-4517400-supplementary.pdf]

Supplemental information

Supplemental Table S1. Details on the sites used, including treatments for each citrus grove

City	County	Grove treatment	Latitude	Longitude	Windbreak species	Windbreak height (m)	Fruit	Treatments
Monticello	Jefferson	Treatment	30°37′38.28″N	83°49′54.34″W	*Thuya* spp.	2.4	Satsuma mandarin	purple coneflower and blanketflower, coral honeysuckle, all flowers, untreated control
Monticello	Jefferson	Control	30°62′48.70″N	83°82′71.86″W	*Quercus* sp.	18	Satsuma mandarin	untreated control
Lake Alfred	Polk	Treatment (Year 1)	28°07'10.7"N	81°42'18.6"W	*Eucalyptus* sp.	13	Sweet orange	blanketflower, coral honeysuckle, buttonbush, all flowers, untreated control
Lake Alfred	Polk	Treatment (Year 2)	28°07'29.6"N	81°42'51.9"W	*Eucalyptus* sp.	13	Sweet orange	blanketflower, coral honeysuckle, American beautyberry, all flowers, untreated control
Lake Alfred	Polk	Control	28° 6' 46.77"N	81° 42' 43.13"W	*Eucalyptus* sp.	13	Sweet orange	untreated control

Supplemental Table S2. Details on the plant species cultivated in this study along with naturally occurring species.

Common name	Plant species	Habit and lifespan	Plant height	Spread	Flower color and size	Fruits	Bloom period	Native distribution
Common blanket flower	*Gaillardia pulchella* (Foug.)	Herb perennial and annual	1-3 ft	20 in	Flowers in shades of red, orange, and yellow and flower heads up to 2 in.	Dark brown, conical seed 1/8-1/6 in. long	Yearround	North America (CO, OK, TX, UT, AZ, south to Jalisco in Mexico)
Purple coneflower	*Echinacea purpurea* (L.) Moench	Herb perennial	1-5 ft	4-20 in	Purple, inflorescence 2.5-6 in	Black achenes, 4-sided, <1 in. long and wide	Summer, Fall	North America (FL, GA, TX…)
Coral honeysuckle	*Lonicera sempervirens* (L.)	Vine perennial	10-20 ft	36-72 in	Red, 2 in	Red to black 0.25 in.	Mid-spring and intermittently thereafter	North America (ME to FL & TX)
Buttonbush	*Cephalanthus occidentalis* (L.)	Shrub Perennial	5 -20 ft	6-8 ft	White, 2 in	Reddish brown woody seeds. 0.5 in	Summer in Florida	Eastern and southern US
American beautyberry	*Callicarpa americana* (L.)	Shrub Perennial	4-6 ft	4-6ft	Pink, 0.5 -1.5 in	Purple seeds, 0.1-0.2 in	Summer	Midwest and Southeast US
Spanish needles	*Bidens alba* (L.)	Annual	1-4 ft	1-3ft	White and yellowish flowers, 0.5 in	Black, 0.5 - 1 in	Yearround	North and South America
Lantana	*Lantana camara* (L.)	Shrub Perennial	3-6 ft	1-3 ft	Orange, pink, white, 2 in	Shiny, green to black, 0.05 in	Yearround	Central and South America
Mexican prickly poppy	*Argemone mexicana* (L.)	Herb Annual	1-3 ft	1 ft	Yellow,orange flowers 1-3 in	1.5 in	Yearround	North America (TX, TN, VA, Fl)
Florida tasselflower	*Emilia fosbergii* Nicolson	Herb Annual	8-24 in		Pink, purple, and red, 0.4 to 0.5 in	Reddish brown to	Yearround	North and South Americas, India,

						white in color, 0.2 in		China, southeast Asia
Florida pusley	*Richardia scabra* L.	Annual	12-18 in		White, star shaped flowers	0.01 in	Yearround	Southeast North America
Common ragweed	*Ambrosia artemisiifolia* L.	Annual	1-5 ft		green flowers, 0.07 - 0.1 in	yellowish or red woody seeds, 0.12-0.16 in	Late summer	North and South America
Evening primrose	*Oenothera laciniata* Hill	Herb Perennial	4-18 in		Yellow to pink flowers, 0.2-0.6 in	0.3 - 1.5 in	Spring, Summer, Fall	Midwest and Eastern US
Pepperweed	*Lepidium virginicum* L.	Annual	4-20 in		White, 0.1 in	0.1 in	Yearround	North America
Southern river sage	*Salvia misella* Kunth	Perennial	0.6-10 in	3-5 ft	Blue, 2.5-3 in		Spring, Summer, Fall	Florida and Caribbean
Pink purslane	*Portulaca Pilosa* L.	Annual	0.6 in	1 ft	Pink and purple, 0.2-0.5 in	Black, 0.01-0.02 in	Spring, Summer, Fall	North and South America
Bitter gourd	*Momordica charantia* L.	Vine Annual	2-3 ft	12-20 ft	Yellow, 1-3 in	Yellow or orange, 4-8 in	Summer	Africa, Asia, Australia. Non-native to Americas.
Passion flower	*Passiflora incarnata* L.	Vine Perennial	12-25 ft	12-25 ft	Purple, pink, or blue, 3 in	Green or yellow, 2 in	Spring, Summer, Fall	Eastern US

Supplemental Table S3. Plant establishment by species, treatment role, location, year, and planting density

**Species**	**Common name**	**Treatment role**	**Location**	**Planting date**	**Density (plants/plot)**	**Notes**
Echinacea purpurea	Purple coneflower	Herbaceous (Flower)	North Florida only	Nov 2020	12	Failed to establish in Central Florida; retained in North Florida Flower treatment
Gaillardia pulchella	Blanket flower	Herbaceous (Flower)	North & Central Florida	Jan 2021	12	Added after purple coneflower failed to establish in Central Florida
Lonicera sempervirens	Coral honeysuckle	Vine	North & Central Florida	Nov 2020 (initial); density increased 2021	7 (2020) → 14 (2021)	Slow to establish in Year 1; density doubled in Year 2
Cephalanthus occidentalis	Buttonbush	Shrub	Central Florida only	Nov 2020	12	Died during dry spring 2021; not replanted (dead structures retained in plots)
Callicarpa americana	American beautyberry	Shrub	Central Florida only	Jan 2022	12	Replacement shrub species for Year 2, planted in the new Central Florida grove

Supplemental Table S4. A summary of the sums, means, and SEs of arthropod predators found in each location, grove type, and row for vacuum sampling. Note that the overall sums are not comparable between locations due to differences in sampling effort.

Sampling method	Location	Grove	Row	n	Mean predator abundance	Predator abundance SE	Mean predator group diversity	Predator richness SE	Sum of predators
Vacuum sampling	North Florida	Control	1	36	1.72	0.33	1.14	0.155	62
		Control	3	36	1.81	0.45	1.03	0.146	65
		Control	6	36	1	0.222	0.75	0.14	36
		Treatment	1	36	1.64	0.376	0.889	0.168	59
		Treatment	3	36	1.5	0.305	0.972	0.167	54
		Treatment	6	36	0.944	0.195	0.722	0.141	34
	Central Florida	Control	1	52	4.06	0.598	2.75	0.122	211
		Control	3	56	3.96	0.605	2.54	0.141	206
		Control	6	52	4.31	0.921	2.46	0.124	224
		Treatment	1	53	10.2	1.25	3.21	0.107	572
		Treatment	3	52	11.2	1.59	3.32	0.145	596
		Treatment	6	52	11.3	1.86	3.15	0.141	586
Yellow sticky traps	North Florida	Control	Windbreak	20	38.7	4.5	4.17	0.286	774
		Control	1	20	19.5	3.65	3.47	0.302	390
		Control	3	20	22.2	3.1	4.34	0.261	445
		Control	6	19	22.6	2.84	3.19	0.233	429
		Treatment	Windbreak	20	68.6	3.55	32.5	0.246	1371
		Treatment	1	20	30	3.8	4.47	0.277	599
		Treatment	3	20	18.9	2.65	3.02	0.209	378
		Treatment	6	20	20.8	2.95	3.66	0.256	416
	Central Florida	Control	Windbreak	49	57.5	1.9	5.27	0.114	2818
		Control	1	52	66.4	1.87	7.06	0.0952	3454
		Control	3	50	64.8	1.78	4.9	0.0823	3238
		Control	6	53	53.4	1.85	5	0.0681	2828
		Treatment	Windbreak	52	96.2	2.15	11	0.0841	5004
		Treatment	1	51	91.3	2.25	13.5	0.0733	4657
		Treatment	3	51	72.6	2	8.55	0.0741	3702
		Treatment	6	52	58.7	2	7.57	0.0824	3051

Supplemental Table S5. A table of the sums of each group of arthropod predators found in each location, grove type, and row for vacuum sampling. Despite including mantids due to their presence in fields, none were collected via vacuum sampling or sticky traps.

Sampling method	Location	Grove	Row	n	Dolicho podidae	Syrphidae	Wasps	Formicidae	Coccinellidae	Araneae	Orius	Chrysopidae	Mantidae
Vacuum samples	North Florida	Control	1	36	6	0	4	5	1	24	2	2	0
		Control	3	36	17	1	7	5	2	19	0	2	0
		Control	6	36	6	0	2	4	4	13	0	1	0
		Treatment	1	36	8	0	8	6	8	21	0	0	0
		Treatment	3	36	6	1	4	3	2	24	0	3	0
		Treatment	6	36	3	0	1	6	7	18	0	1	0
	Central Florida	Control	1	52	32	0	33	92	0	51	0	3	0
		Control	3	56	49	0	11	87	2	55	0	2	0
		Control	6	52	35	1	18	131	2	36	0	1	0
		Treatment	1	53	65	0	44	341	4	107	3	8	0
		Treatment	3	52	120	0	51	299	5	107	8	6	0
		Treatment	6	52	121	0	59	299	2	97	6	2	0
Sticky traps	North Florida	Control	W	20	319	3	54	261	7	36	6	0	0
		Control	1	20	225	3	27	82	4	14	3	2	0
		Control	3	20	333	6	18	53	2	11	6	0	0
		Control	6	19	321	0	18	52	3	7	2	0	0
		Treatment	W	20	446	7	45	792	5	11	0	0	0
		Treatment	1	20	390	1	42	89	1	19	2	0	0
		Treatment	3	19	279	2	18	52	3	5	0	0	0
		Treatment	6	20	284	0	21	53	2	7	3	0	0
	Central Florida	Control	W	52	1421	0	814	384	10	181	8	1	0
		Control	1	51	1478	0	898	947	8	117	4	2	0
		Control	3	51	1467	0	820	850	5	95	1	0	0
		Control	6	52	1136	0	949	617	5	121	0	0	0
		Treatment	W	49	2828	1	993	899	6	264	13	0	0
		Treatment	1	52	2000	0	1018	1313	7	303	16	0	0
		Treatment	3	50	1462	0	709	1296	5	225	6	0	0
		Treatment	6	53	1293	0	603	913	10	227	5	0	0

Supplemental Table S6. Estimated parameters, standard errors, z-statistics, and p-values for the abundance of each predator group in treatment groves compared with control groves, presented by sampling method and location.

Sampling Method	Location	Predators	Orchard	Estimate	Std. Error	z value	p-value
Sticky trap	North Florida	All Predators	Intercept	3.770	0.206	18.339	<0.001
			Treatment vs control	0.136	0.102	1.335	0.182
		Araneae	Intercept	0.315	0.287	1.096	0.273
			Treatment vs control	-0.452	0.247	-1.829	0.067
		Coccinellidae	Intercept	-1.181	0.421	-2.804	0.005
			Treatment vs control	-0.372	0.392	-0.949	0.343
		Dolichopidae	Intercept	2.723	0.264	10.307	<0.001
			Treatment vs control	0.118	0.106	1.109	0.267
		Formicidae	Intercept	2.906	0.378	7.675	<0.001
			Treatment vs control	0.057	0.281	0.203	0.839
		Orius sp.	Intercept	-1.882	0.556	-3.382	0.001
			Treatment vs control	-1.255	0.509	-2.464	0.014
		Syrphidae	Intercept	-2.918	1.094	-2.668	0.008
			Treatment vs control	-0.169	0.552	-0.306	0.760
		Wasps	Intercept	1.585	0.247	6.426	<0.001
			Treatment vs control	0.197	0.141	1.402	0.161
	Central Florida	All Predators	Intercept	4.085	0.190	21.470	<0.001
			Treatment vs control	0.117	0.047	2.500	0.012
		Araneae	Intercept	0.944	0.158	5.976	<0.001
			Treatment vs control	0.703	0.073	9.687	<0.001
		Coccinellidae	Intercept	-1.976	0.354	-5.586	<0.001
			Treatment vs control	-0.002	0.285	-0.007	0.995
		Dolichopidae	Intercept	3.101	0.412	7.521	<0.001
			Treatment vs control	-0.024	0.079	-0.302	0.763
		Formicidae	Intercept	1.349	0.449	3.003	0.003
			Treatment vs control	0.230	0.090	2.568	0.010
		Orius sp.	Intercept	-4.339	1.111	-3.905	<0.001
			Treatment vs control	1.225	0.360	3.400	<0.001
		Wasps	Intercept	2.843	0.135	21.060	<0.001
			Treatment vs control	-0.023	0.062	-0.371	0.711
	North Florida	All Predators	Intercept	0.435	0.227	1.911	0.056
			Treatment vs control	-0.116	0.156	-0.746	0.456
		Araneae	Intercept	-1.469	0.379	-3.879	<0.001
			Treatment vs control	0.000	0.263	0.000	1.000
		Coccinellidae	Intercept	-1.228	0.888	-1.383	0.167
			Treatment vs control	0.794	0.539	1.473	0.141
		Dolichopidae	Intercept	-2.535	1.036	-2.447	0.014
			Treatment vs control	-0.712	0.611	-1.165	0.244

Vaccum		Formicidae	Intercept	-1.744	0.700	-2.491	0.013
			Treatment vs control	0.081	0.384	0.212	0.832
		Wasps	Intercept	-0.890	0.488	-1.824	0.068
			Treatment vs control	-0.168	0.213	-0.788	0.430
	Central Florida	All Predators	Intercept	1.368	0.171	8.008	<0.001
			Treatment vs control	0.889	0.107	8.290	<0.001
		Araneae	Intercept	-0.115	0.220	-0.523	0.601
			Treatment vs control	0.591	0.153	3.853	0.000
		Coccinellidae	Intercept	-2.744	1.259	-2.179	0.029
			Treatment vs control	0.963	0.623	1.546	0.122
		Dolichopidae	Intercept	-0.919	0.374	-2.459	0.014
			Treatment vs control	0.707	0.237	2.980	0.003
		Chrysopidae	Intercept	-3.191	0.602	-5.304	<0.001
			Treatment vs control	0.892	0.509	1.754	0.079
		Formicidae	Intercept	0.619	0.227	2.727	0.006
			Treatment vs control	0.906	0.144	6.301	<0.001
		Wasps	Intercept	-0.896	0.302	-2.966	0.003
			Treatment vs control	0.737	0.227	3.240	0.001

Supplemental Table S7. Floral visitation frequency of predatory/natural enemy arthropod taxa on flowering plant species at Central Florida (CREC) and North Florida (NFREC) field sites.

Values show the number of observed visits by each arthropod taxon to each plant species, with the percentage of that plant's total recorded visits shown in parentheses. Data are the same observations summarized in the Sankey diagram (Fig. 4) and are provided here to allow quantitative comparison across plant × taxon combinations. Percentages are calculated within each plant (row-wise, i.e., relative to that plant's total visit count). Total sample sizes were n = 400 visits at Central Florida and n = 260 visits at North Florida. Underlying observation-level data are provided in the 'Raw data' sheet.

**Location**	**Plant**	**Ant**	**Syrphidae**	**Wasp**	**Spider**	**Cantharidae**	**Coccinellidae**	**Orius sp.**	**Miridae**	**Dragonfly**	**Total visits**
Central Florida	Blanket flower	150 (86.7%)	2 (1.2%)	6 (3.5%)	12 (6.9%)	0	1 (0.6%)	2 (1.2%)	0	0	**173**
Central Florida	Spanish needles	12 (11.0%)	57 (52.3%)	26 (23.9%)	9 (8.3%)	4 (3.7%)	1 (0.9%)	0	0	0	**109**
Central Florida	Florida pusley	68 (95.8%)	2 (2.8%)	1 (1.4%)	0	0	0	0	0	0	**71**
Central Florida	Bitter gourd	26 (96.3%)	0	1 (3.7%)	0	0	0	0	0	0	**27**
Central Florida	Lantana	6 (60.0%)	0	1 (10.0%)	3 (30.0%)	0	0	0	0	0	**10**
Central Florida	Ragweed	1 (20.0%)	0	2 (40.0%)	2 (40.0%)	0	0	0	0	0	**5**
Central Florida	Tassel flower	0	1 (25.0%)	2 (50.0%)	1 (25.0%)	0	0	0	0	0	**4**
Central Florida	Coral honeysuckle	1 (100.0%)	0	0	0	0	0	0	0	0	**1**
North Florida	Wild mustard	0	114 (82.0%)	0	0	0	22 (15.8%)	0	3 (2.2%)	0	**139**
North Florida	Blanket flower	0	24 (31.2%)	16 (20.8%)	7 (9.1%)	0	15 (19.5%)	0	14 (18.2%)	1 (1.3%)	**77**
North Florida	Common vetch	0	7 (30.4%)	0	0	0	16 (69.6%)	0	0	0	**23**
North Florida	Dead nettle	0	7 (77.8%)	1 (11.1%)	1 (11.1%)	0	0	0	0	0	**9**
North Florida	Black medic	0	4 (100.0%)	0	0	0	0	0	0	0	**4**
North Florida	Purple Coneflower	0	2 (50.0%)	2 (50.0%)	0	0	0	0	0	0	**4**
North Florida	Florida pusley	0	1 (100.0%)	0	0	0	0	0	0	0	**1**
North Florida	Coral honeysuckle	0	0	0	0	0	0	0	0	1 (100.0%)	**1**
North Florida	Pickle weed	0	1 (100.0%)	0	0	0	0	0	0	0	**1**
North Florida	Spanish needles	0	1 (100.0%)	0	0	0	0	0	0	0	**1**
**Central Florida**	**All plants (site total)**	**264 (66.0%)**	**62 (15.5%)**	**39 (9.8%)**	**27 (6.8%)**	**4 (1.0%)**	**2 (0.5%)**	**2 (0.5%)**	**0**	**0**	**400**
**North Florida**	**All plants (site total)**	**0**	**161 (61.9%)**	**19 (7.3%)**	**8 (3.1%)**	**0**	**53 (20.4%)**	**0**	**17 (6.5%)**	**2 (0.8%)**	**260**

Note: '0' indicates the taxon was not observed on that plant. Blank combinations were not structurally possible (e.g., Cantharidae and Orius sp. were only recorded at Central Florida; Miridae and Dragonfly only at North Florida).

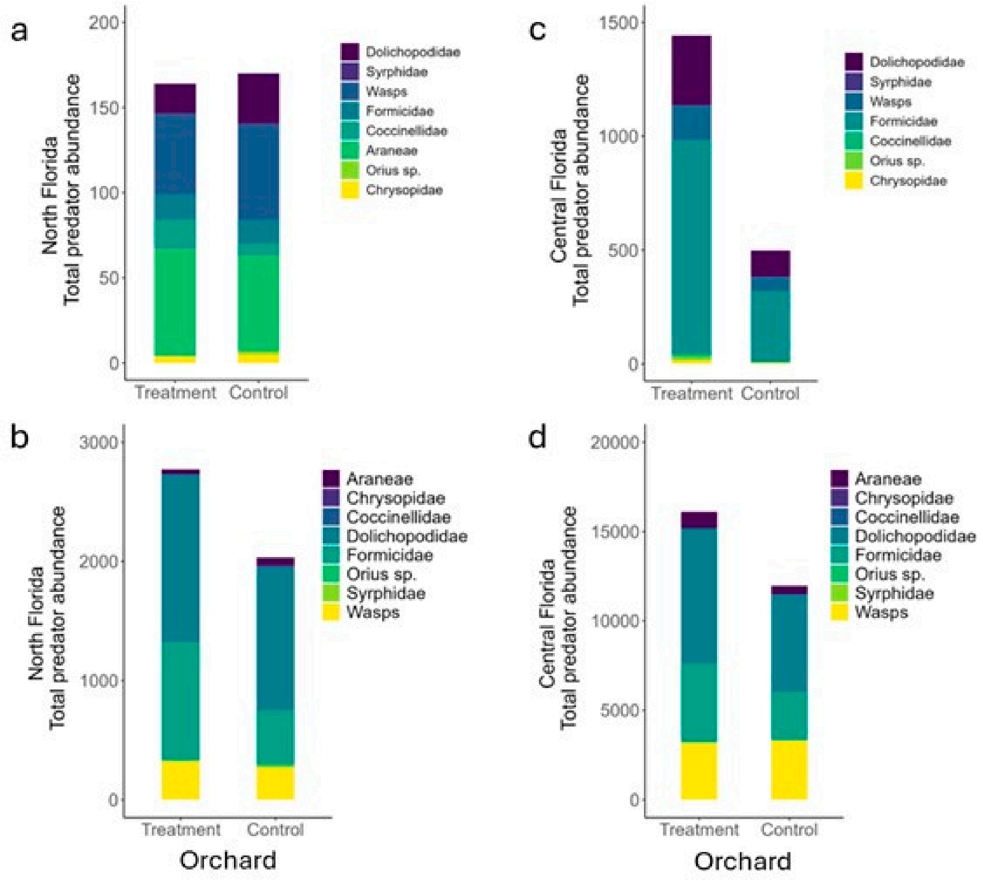

Supplemental Figure S1. Proportional composition of predatory arthropod groups collected via vacuum (a, c) and sticky trap (b, d) sampling in North Florida (a, b) and Central Florida (c, d) from 2021 to 2023.

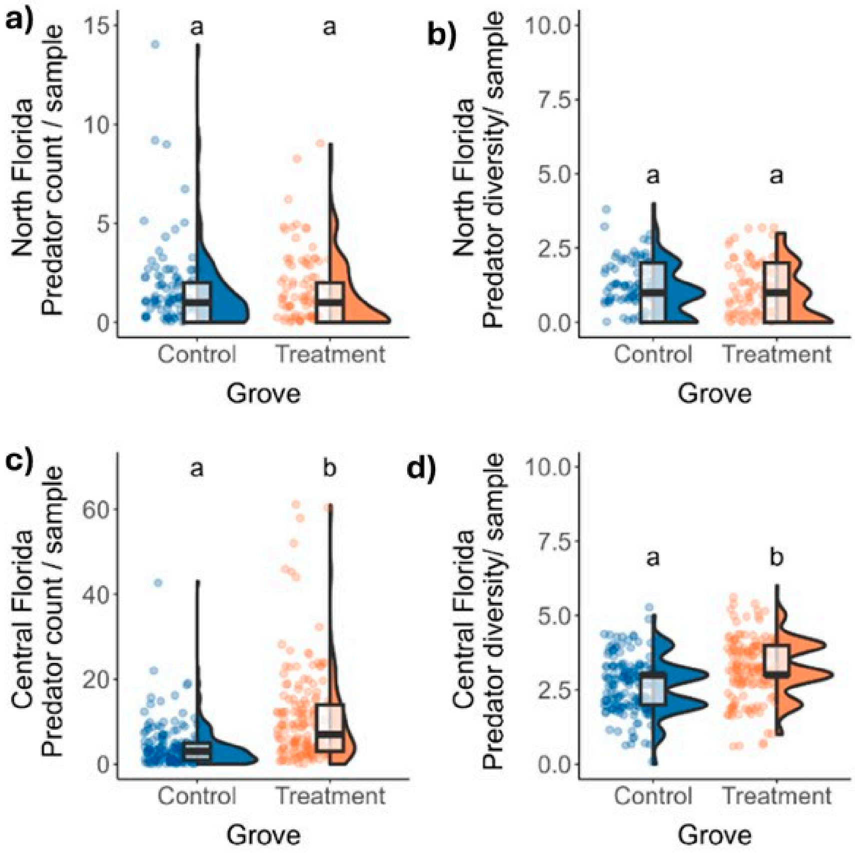

Supplemental Figure S2. Data points, density kernel plots, and boxplots showing the distribution of a) predator abundance and, b) group diversity in north Florida, as well as c) predatory abundance and d) group diversity in central Florida from vacuum samples. Data points have been jittered to aid in visualization since many values are identical.

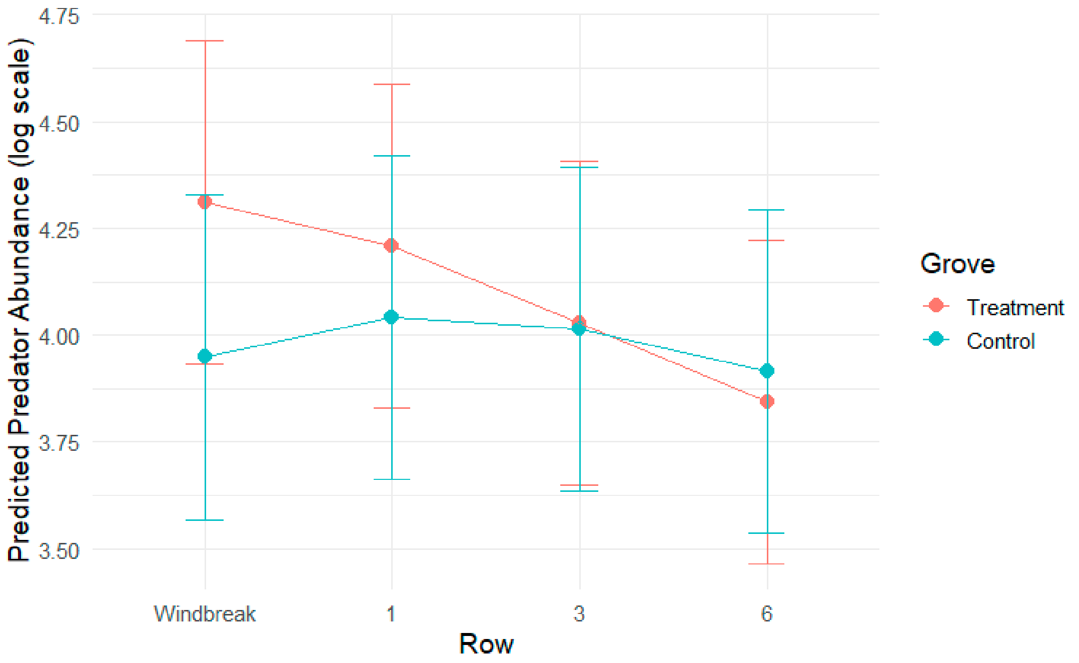

Supplemental Figure S3. Interaction between grove treatment and distance from windbreak on predicted predator abundance

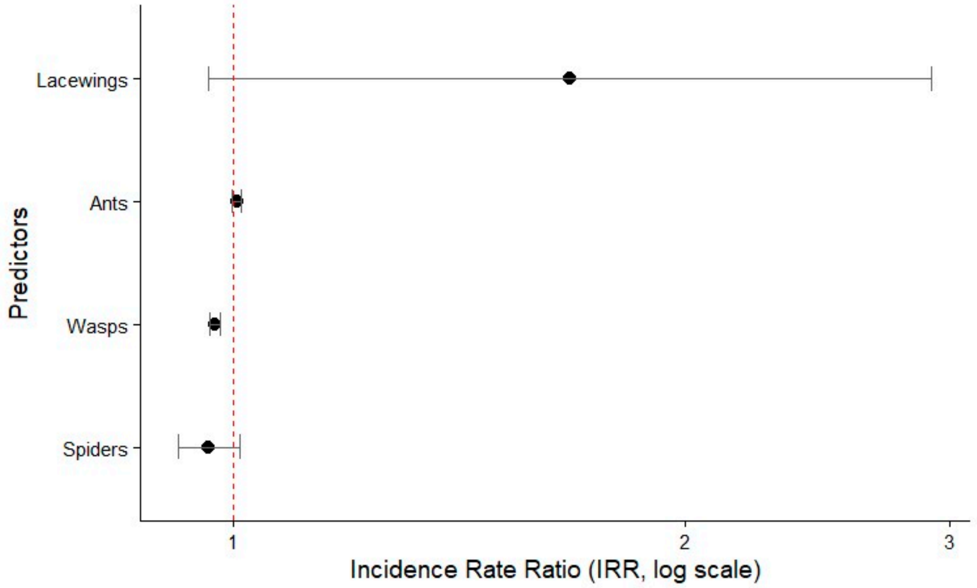

Supplemental Figure S4. Incidence rate ratios (IRRs) for the association between natural enemy abundances (wasps, spiders, ants, and lacewings) and Asian citrus psyllid (*Diaphorina citri*) abundance in Central Florida citrus groves. Error bars represent 95% confidence intervals. An IRR > 1 indicates a positive co-occurrence (higher natural enemy counts corresponding to higher ACP counts), whereas an IRR < 1 indicates an inverse association. Among the evaluated

groups, wasps showed a statistically significant inverse association with ACP abundance (IRR = 0.973, 95% CI: 0.966–0.981, $p < 0.001$). Spiders (IRR = 0.964, 95% CI: 0.919–1.010, p = 0.123), ants (IRR = 1.006, 95% CI: 0.999–1.013, p = 0.090), and lacewings (IRR = 1.677, 95% CI: 0.962–2.920, p = 0.068) showed no statistically significant associations with ACP counts at the $\alpha = 0.05$ level.
